# Academic achievement and emotional and behavioural problems: The moderating role of gender

**DOI:** 10.1177/13591045211059410

**Published:** 2022-02-03

**Authors:** Pedro Dias, Lurdes Veríssimo, Alexandra Carneiro, Bárbara Figueiredo

**Affiliations:** 1 Universidade Católica Portuguesa, Research Centre for Human Development, Porto, Portugal; 2 Faculty of Education and Psychology, 59207 Universidade Católica Portuguesa, Porto, Portugal; 3 Psychology Research Center, School of Psychology, 56059 University of Minho, Braga, Portugal

**Keywords:** Academic achievement, emotional and behavioural problems, children and adolescents, parents, teachers, gender, CBCL, TRF

## Abstract

The present study aimed to explore the association between academic achievement and emotional and behavioural problems and the moderation role of gender in this association. 1350 Portuguese school-aged children and adolescents from first to ninth grade (6–15-year-old), part of a national representative sample, were assessed by teachers and parents with questionnaires from the Achenbach System of Empirically Based Assessment (ASEBA). Results show that academic achievement significantly predicts child and adolescent’s internalizing, externalizing, and total problems. Gender moderates the association between academic achievement and child and adolescent’s externalizing and total problems, both at school and in the family context. The results underscore the relevance of academic achievement in children and adolescent’s emotional and behavioural problems, and particularly in boys.

## Introduction

Academic achievement (AA) plays a central role in children and adolescents’ developmental trajectories, determining life opportunities considering its impact in the development of autonomy, relatedness and competence (Ryan & Deci, 2009; Steinmayr et al., 2016). Emotional and Behavioural Problems (EBPs) have received particular attention in the AA literature over the last decades, since between 10 and 50% of school-aged children with externalizing behaviours present low AA (Hinshaw, 1992).

The association between AA and emotional (internalizing problems—IP) and behavioural (externalizing problems—EP) problems has been conceptualized through two competing perspectives. On the one hand, several authors have highlighted the role of EBP in AA, assuming that difficulties in self-regulated behaviour and emotions contribute to impaired performance at school (e.g. Brennan et al., 2012; McLeod et al., 2012; Saleem & Mahmood, 2012; Sijtsema et al., 2014). On the other hand, with less prominence, some authors focused on low AA as one possible cause of EBP in children and adolescents (e.g. Verboom et al., 2014; Zimmermann et al., 2013). This perspective, labelled by Moilanen et al. (2010) as the *academic incompetence hypothesis*, suggests that early problems in academic performance could prompt or exacerbate internalizing or externalizing symptoms.

### Influence of emotional and behavioural problems on academic achievement

Several studies support the influence of EBP on children and adolescents’ AA (Jaycox et al., 2009; McLeod et al., 2012; Roeser et al., 1998; Sijtsema et al., 2014). Adolescents with high levels of depression, attention problems and delinquency score usually lower on standardized achievement tests (Roeser, et al., 1998). The negative effects of depressive symptoms lead to a decline in school results and overall poor academic performance (Jaycox et al., 2009). Attention problems, delinquency and substance abuse are also strongly associated with low AA, and youth who experienced two or more problems had lower AA than youth who experienced only one problem (McLeod et al., 2012). Sijtsema et al. (2014) found that the strongest predictor of poor academic performance in primary and secondary school, adjusting for comorbidity, was attention problems, as reported by parents and teachers. The same study revealed that all EP were negatively associated with academic performance, except for self-reported conduct problems in primary school aged females. Differences were found in the explained variance of academic performance based on the informant: Most variance was explained by teachers’ reports of psychopathology (36.8%), followed by parents’ reports (16.0%) and children’s reports (2.4%) (Sijtsema et al., 2014).

### Influence of academic achievement on emotional and behavioural problems

Research has also focused the inverse process, considering the influence of low AA on EBP. The competency-based model of depression describes the mechanism underlying the relationship between poor performance in several domains of functioning and depressive problems (Cole, 1991). This model emphasizes that children who perform poorly in one or more functioning domains, such as the academic domain, may receive negative feedback from others, promoting negative self-perception and subsequently triggering depressive problems. Verboom et al. (2014) concluded that, regardless of gender, there is a considerable stability in the association between depressive and academic problems over time. In the same longitudinal study, lower academic performance was correlated with higher levels of depressive problems and a decrease in academic performance was associated with an increase in depressive problems. In addition, several studies have found that high levels of academic performance can protect against depressive problems (e.g. Lazaratou et al., 2010; Qin et al., 2021).

Considering EP, Miles and Stipek (2006) concluded that poor AA predicts aggressive behaviour. When children or adolescents repeatedly face an inability to achieve valued academic goals, they may become unhappy or annoyed, leading to the emergency of EP. The negative feedback of poor grades may be a powerful agent that, however, does not decrease children or adolescents’ behaviour problems, and instead seems to lead to an exacerbation of these problems (Zimmermann et al., 2013). This longitudinal study also emphasized that school grades consistently predicted more severe subsequent EP over time (Zimmermann et al., 2013).

Despite many years of research, empirical evidence on the association between EBP and AA is limited (e.g. Kulkarni et al., 2020), and few studies have considered: (1) multiple problems simultaneously, which may produce biased estimates (Sijtsema et al., 2014), (2) informant-specific associations between EBP and school functioning (Sijtsema et al., 2014), and (3) AA as a predictor of EBP (Verboom et al., 2014); (4) the role of gender in the association between AA and EBP (e.g. Moilanen et al., 2010). Additionally, as much as we know, no study has addressed the association between children and adolescents EBP and AA or the reverse, in the Portuguese context.

Using a representative sample of Portuguese school-aged children and adolescents, the present study aimed to explore the association between AA and EBP, assessed by teachers and parents. Under a developmental psychopathology approach, the moderation role of gender was also considered in this association. Gender differences have being consistently reported in child psychopathology literature (e.g. Lazaratou et al., 2010; Rutter & Sroufe, 2000; Somers et al., 2020). In a study involving participants from 44 societies (including Portugal), with data from more than 60,000 children and adolescents assessed with the Child Behavior Checklist (CBCL) and more than 37,000 children and adolescents assessed with the Teacher Report Form (TRF), authors found gender effects on both the CBCL and TRF. However, girls tended to present higher scores than boys on Internalizing, whereas boys tended to have higher scores than girls on EP across the two instruments (Rescorla et al., 2012).

## Method

### Participants

The study included 1350 children and adolescents (47.4% female) with a mean age of 10.06 years (*SD* = 2.83, range 6–15 years). Participants attended public and private schools, from first to ninth grade. This sample was drawn from a larger study examining the assessment of psychopathology and the validation of the ASEBA preschool and school-age forms among Portuguese children and adolescents aged between 1-½ and 18 years (nationally representative sample; *N* = 3437) (cf. Achenbach, et al., 2014). The measures were mostly responded by mothers (77.4%), but also by fathers (16.3%) or other relatives (6.3%). Family SES ranged from low to high (M = 2.96; *SD* = 1.30), based on father’s profession (using an adaptation of Graffar’s five-point scale; Amaro, 2001). Fathers’ age ranged from 23 to 74 years (M = 41.73; *SD* = 6.26). Mothers’ age ranged from 21 to 73 years (M = 39.38; *SD* = 5.78). TRF was responded by the teacher responsible for the student’s class. On average, teachers reported knowing the student for 8.08 months (*SD* = 8.30) and stated that they knew them well (M = 2.17; *SD* = .58, on a 3-point scale ranging from 1 – not well – to 3 – very well).

### Measures

*Achenbach System of Empirically Based Assessment – Child Behavior Checklist 6–18* (ASEBA-CBCL; Achenbach, et al., 2014). The CBCL consists of 118 items that describe behavioural and emotional problems in children and adolescents aged between 6 and 18 years. Parents are requested to rate child’s functioning in the last 6 months using a Likert scale anchored with the descriptors: 0 (not true), 1 (somehow or sometimes true) and 2 (very true or often true).

*Achenbach System of Empirically Based Assessment – Teacher Report Form* (ASEBA-TRF; Achenbach, et al., 2014). Teachers are requested to rate children and adolescents based on their functioning in the last 2 months, using a Likert scale anchored with the descriptors: 0 (not true), 1 (somehow or sometimes true) and 2 (very true or often true). TRF also collects information on child’s and adolescent’s academic achievement on a scale ranging from 1 (very low than expected level) to 5 (much higher than expected level).

Ninety-three of both instruments’ items are similar (e.g. cries a lot; fearful; argues a lot). In the CBCL, the remaining items are related to behaviour at home (e.g. nightmares; drinks alcohol), and, in the TRF, the remaining items are related to behaviour in school (e.g. difficulty following instructions, cause disturbances among peers).

Both CBCL and TRF provide information on internalizing and EP, based on lower-level empirically based syndromes. IP score includes three syndrome scales: anxious/depressed, withdrawn/depressed, and somatic complaints. EP score includes two syndrome scales: rule-breaking behaviour and aggressive behaviour. Total problems (TP) score includes, in addition to internalizing and EP, results from three other syndrome scales (social problems, thought problems and attention problems) and other problems.

Psychometric properties of CBCL and TRF were assessed, in the larger validation study, through confirmatory factor analysis, internal consistency analysis (Cronbach’s Alpha) and group difference analysis (clinical vs. non-clinical samples). The psychometric analyses support the use of the Portuguese version of these instruments, presenting adequate validity and reliability (Achenbach et al., 2014). In the Portuguese normative sample of children aged between 6 and 18 years old, *alpha* coefficients were adequate (CBCL: IP α = .85, EP α = .88, and TP α = .96; TRF: IP α = .83, EP α = .91, and TP α = .95). Confirmatory factor analysis supported the original factor structure of these instruments for the Portuguese sample (Achenbach, et al., 2014).

### Procedures

The data collection procedure complied with legal and ethical requirements (authorizations from the Ministry of Education and Data Protection Authority in Portugal were obtained). Schools were contacted and authorization was given by the direction/pedagogical boards of schools. Parents of randomly selected children and adolescents received information about the study goals as well as the procedures, and written consent was obtained. Parents who gave consent received the CBCL and returned the instrument by mail. TRF were responded by teachers who spent more time with the children; responses were sent to the research team by mail. Participants’ anonymity was assured by using codes for each participant, previously assigned to the envelopes and questionnaires.

### Analytic strategy

All analyses were performed using SPSS 24.0 (IBM Corp, 2013). Children were excluded if ratings were missing for more than eight problem items on CBCL or TRF, as recommended by Achenbach et al. (2014); in the remaining participants, the missing items were replaced by mean. Considering the sample size, the number of participants with eight or more items missing was residual (CBCL, *N* = 1; TRF, *N* = 0).

AA was calculated based on TRF’s information. The mean of the main three school subjects (e.g. Portuguese, Mathematics, Sciences) was calculated for each child or adolescent, based on the five-point scale (M = 3.41; *SD* = .88). .

The association between AA and EBP was examined using Pearson correlations and the association between gender and EBP was examined with point-biserial correlations. Multiple linear regressions were carried out. In order to examine the individual contribution of AA on emotional and behavioural functioning, while controlling for child gender, the *R*^2^ change was calculated. Finally, and to examine the role of gender as a moderator of the relation between AA and EBP, model 3 PROCESS macro, a computational tool for SPSS (Hayes, 2013) was used.

## Results

### Association between academic achievement and internalizing, externalizing and total problems

Academic achievement was negatively correlated to parents’ and teachers’ reported internalizing, externalizing and TP in children and adolescents (see Table 1). Regarding gender, boys tend to present higher scores in externalizing and TP assessed by parents and by teachers (see Table 1).

**Table 1.** Correlation between ASEBA scales and academic achievement (*N* = 1350).

			AA	Gender (r_pb_)
			Total sample	Total sample
ASEBA scales				
CBCL	Internalizing		−.11**	.02
	Externalizing		−.17**	−.07**
	Total		−.22**	−.09**
TRF	Internalizing		−.09**	.01
	Externalizing		−.24**	−.17**
	Total		−.34**	−.16**

*p < .050, **p < .010.

### The contribution of gender and academic achievement to internalizing, externalizing and total problems

Regarding parents’ reports, gender predicted externalizing and TP. In both cases, gender explains 1% of the total variance. However, when AA is added to the model, it predicts internalizing, externalizing and TP. These results indicate that AA is a predictor of interest to understand EBP, given that, for IP, it adds 1% to the explained variance in EBP, and it also adds 3% to the explained variance for EP and 5% for TP assessed by parents (see Table 2).

**Table 2.** Predictors of internalizing, externalizing and total problems in CBCL and in TRF.

		CBCL						TRF					
		*B*	*T*	R2 (adj)	F (df)	R2	F	*B*	*t*	R2 (adj)	F (df)	R2	F
						Change	Change					Change	Change
Internalizing problems	Step 1												
	Gender	.23	.69	.00 (.00)	.47*** (1349)	.00	.47	.11	.40	.00 (.00)	.16 (1349)	.00	.16
	Step 2												
	Gender	.28	.84	.01 (.01)	8.76*** (1349)	.01	17.05***	.15	.57	.01 (.01)	10.53*** (1349)	.01	20.89***
	AA	−.77	−4.13***					-.67	−4.57***				
Externalizing problems	Step 1												
	Gender	−1.09	−3.36***	.01 (.01)	11.31*** (1349)	.01	11.31***	−1.89	−6.18***	.03 (.03)	38.13*** (1349)	.03	38.13***
	Step 2												
	Gender	−1.01	−3.17**	.04 (.04)	30.06*** (1349)	.03	48.41***	−1.80	−6.02***	.07 (.07)	53.48*** (1349)	.05	66.96***
	AA	−1.25	−6.96***					−1.38	−8.18***				
Total problems	Step 1												
	Gender	−2.68	−2.69**	.01 (.01)	7.22** (1349)	.00	.01**	−5.46	−6.03***	.03 (.03)	36.30*** (1349)	.03	.36.30***
	Step 2												
	Gender	−2.38	−2.45*	.06 (.05)	39.16*** (1349)	.05	70.74***	−5.06	−5.92***	.14 (.13)	105.81*** (1349)	.11	170.75***
	AA	−4.63	−8.41***					−6.32	−13.07***				

*p ≤ .05; **p ≤ .01; ***p ≤ .001.

In the case of teachers, results were very similar to those that were found to parents reports of EBP. Gender predicted EP and TP. In both cases, gender explains 3% of the total variance of these problems. When AA is added to the model, it predicts IP, EP and TP. These results indicate that AA is a predictor that contributes significantly to explain IP because it adds 1% to the explained variance of the model, adds 5% to explain EP and 11% to explain TP assessed by teachers (see Table 2).

### The moderation role of gender in the relation between academic achievement and internalizing, externalizing and total problems

**Figure 1.**
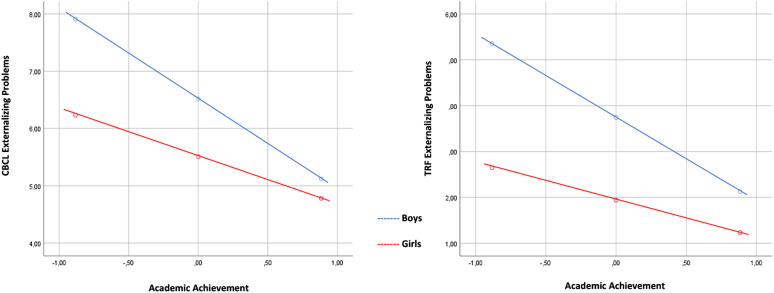
Plot of the interaction between child gender and academic achievement in relation to child’s externalizing problems reported on CBCL and on TRF.

**Figure 2.**
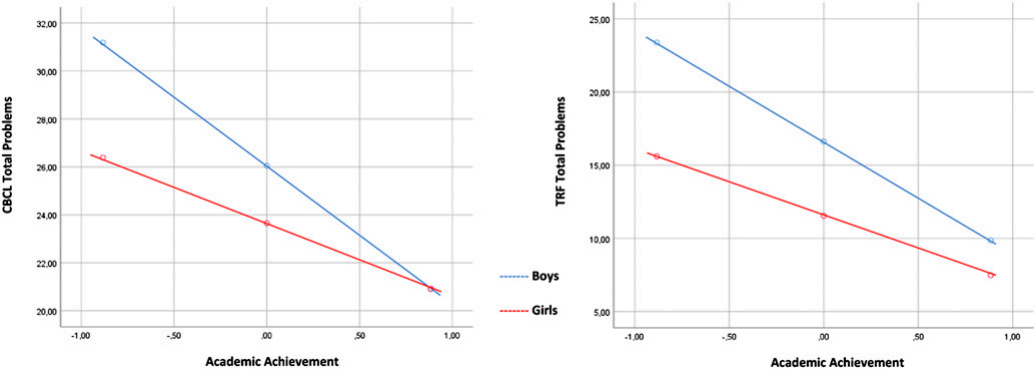
Plot of the interaction between child gender and academic achievement in relation to child’s total problems reported on CBCL and on TRF.

Gender is a moderator in the relation between AA and EP and TP measured using parents report (*R*^2^ change = .003, *F* = 4.39 (1346), *p* = .036; *R*^2^ change = .004, *F* = 5.99 (1346), *p* = .015, respectively) (see Table 3). In both cases there is a significant probability of the effect be observed in the population because zero was not included in the CIs, indicating that the moderator role of gender is significantly different from zero at *p* < .050 (two tailed). AA has a larger impact in externalizing and TP in boys, when compared to girls (*β* = −1.58, *SE* = .24, *t* = −6.62, *p* ≤ .000, LLCI = −2.05, UPCI = −1.11 vs. *β* = −.82, *SE* = .27, *t* = −3.02, *p* = .003, LLCI = −1.35, UPCI = −.29; *β* = −5.81, *SE* = .73, *t* = −7.95, *p* ≤ .000, LLCI = −7.25, UPCI = −4.38 vs. *β* = −3.10, *SE* = .83, *t* = −3.72, *p* ≤ .001, LLCI = −4.73, UPCI = −1.47, respectively) as observed in Figures 1 and 2. In the case of IP, the model is marginally significant (*R*^2^ change = .002, *F* = 2.83 (1346), *p* = .093), and zero was included in the CIs, indicating that the moderator role of gender is not significantly different from zero at *p* < .050 (two tailed).

**Table 3.** Gender moderation role in the relation between academic achievement and internalizing, externalizing and total problems in CBCL and in TRF.

		CBCL							TRF						
		*Unstandardized Coefficient*						95% CI	*Unstandardized Coefficient*						95% CI
		*β*	*SE*	*t*	*R*^2^	*F* (df)	Lower	Upper	*β*	*SE*	*t*	*R*^2^	*F* (df)	Lower	Upper
Internalizing problems	AA	−.74	.19	−3.97***	.01	6.79*** (1346)	−1.11	-.38	-.66	.15	−4.51***	.02	7.10*** (1346)	−.95	−.38
	Gender	.27	.33	.83			−.37	.92	.15	.26	.57			−.36	.66
	Gender*AA	−.63	.37	1.68^+^			−.10	1.37	.15	.30	.52			−.43	.74
Externalizing problems	AA	−1.22	.18	−6.76***	.05	21.55*** (1346)	−1.57	-.86*	−1.34	.17	−7.92***	.08	38.93*** (1346)	−1.67	−1.11
	Gender	−1.01	.32	−3.18**			−1.63	-.39	−1.80	.30	−6.06***			−2.39	−1.22
	Gender*AA	.76	.36	2.10*			.05	1.47	1.03	.34	3.03**			.36	1.69
Total problems	AA	−4.52	.55	−8.19***	.06	28.20*** (1346)	−5.60	−3.44	−6.19	.48	−12.80***	.14	74.27*** (1346)	−7.14	−5.24
	Gender	−2.40	.97	−2.47*			−4.31	−.49	−5.08	.85	−5.96***			−6.75	−3.40
	Gender*AA	2.71	1.11	2.45*			.54	4.89	3.05	.97	3.13**			1.14	4.95

*p < .05; **p < .01; ***p ≤ .001.

Gender is a moderator in the relation between AA and EP and TP measured using teachers report (*R*^2^ change = .006, *F* = 9.18 (1346), *p* = .003; *R*^2^ change = .006, *F* = 9.81 (1346), *p* = .002, respectively) (see Table 3). In both cases there is a significant probability of the effect be observed in the population because zero was not included in the CIs, indicating that the moderator role of gender is significantly different from zero at *p* < .050 (two tailed). In the case of EP, AA has a larger impact for boys than for girls (*β* = −1.83, *SE* = .22, *t* = −8.17, *p* ≤ .000, LLCI = −2.27, UPCI = −1.39 vs. *β* = −.80, *SE* = .26, *t* = −3.14, *p* = .002, LLCI = −1.30, UPCI = −.30, respectively), as observed in Figure 1. For TP there is a similar effect for boys and girls (*β* = −7.65, *SE* = .64, *t* = −11.9, *p* ≤ .001, LLCI = −8.91, UPCI = −6.39 vs. *β* = −4.60, *SE* = .73, *t* = −6.30, *p* ≤ .000, LLCI = −6.04, UPCI = −3.17, respectively), as shown in Figure 2. In the case of IP, the moderation effect is not significant (*R*^2^ change = .000, *F* = .27 (1346), *p* = .601).

## Discussion

The association between AA and EBP has been proposed in the literature. However, few studies focus on AA as a predictor of psychopathological symptoms in school-aged children or adolescents, and the moderating role of child and adolescent’s gender in this association was not yet considered. The present study aimed to explore the association between AA and EBP, assessed by teachers and parents, including the moderation role of gender in this association.

AA was negatively associated with IP, EP, and TP, assessed by both parents and teachers. Results show that gender is a significant predictor of EP and TP assessed by parents and teachers, with boys presenting more problems than girls, as reported by previous research (e.g. Rescorla et al., 2012). When AA was added to the regression model, the percentage of explained variance was significantly higher, also accounting for IP.

These results underscore the importance of academic performance when studying EBP. In fact, school performance has a generalized influence in psychopathological symptoms from 6 to 15 years old, consistently with previous research (Sijtsema et al., 2014; Weidman, Augustine et al., 2015; Zhang et al., 2019). Furthermore, the results provide additional support to the academic incompetence hypothesis (Moilanen et al., 2010), while showing that problems in academic performance impact internalizing and externalizing symptoms.

Considering the influence of gender, and the larger contribution of AA in predicting EP and TP in our sample, a moderation hypothesis was tested, using gender as the moderator of the association between AA and EBP. Results evidence that gender moderates the relation between AA and EP and TP assessed by both parents and teachers, with AA having a higher impact in boys’ EP and TP, when compared to girls. This result highlights the increased risk for boys with low AA in terms of EBP. Boys seem to be more reactive to the effect of low AA, which could be understood considering several developmental dimensions, such as the fact that boys tend to show poorer sociodevelopmental adjustment in school-age period, when compared to girls (e.g. Gresham & Elliott, 1990; Merrell & Gimpel, 2014). This may undermine the future development of skills, such as impulse self-control or adequate cognitive-shifting, related to both academic performance and behaviour regulation.

The moderation effect is shown considering the assessment of both parents and teachers, reinforcing the idea that low AA may be a widespread risk for boys (Moilanen et al., 2010). Indeed, a consistent pattern of results is observed in family and school contexts, highlighting the relevance of these findings, considering these are the central contexts of children and adolescents’ life and development.

### Strengths, limitations and future research

The present study addressed some relevant flaws in the literature, namely by considering the presence of multiple problems, instead of focusing on a single type of problem (Sijtsema et al., 2014). The present study assumed the analysis of AA as a factor affecting EBP (Verboom et al., 2014), which has been significantly less studied than the approach that highlights the influence of EBP in AA. The nature of the sample – representative national sample, including students from public and private schools and different socioeconomic backgrounds – is also a strength of this study, allowing to consider the study’s results as a robust contribution to the literature on this area. Furthermore, the assessment of EBP using parents’ and teachers’ reports allowed to examine these problems in the most relevant contexts of children and adolescents’ life, rather than focusing only on one of these contexts, assuming that AA may impact EBP in both contexts. Finally, the role of gender, as a possible moderator in the relation between AA and EBP was tested, contributing to the scarce literature on this specific developmental variable.

Nevertheless, other variables may have a potential value in the study of the association between AA and EBP. For example, socio-emotional skills (e.g. self-esteem, self-control) and individual cognitive antecedents (e.g. inattention), could be respectively considered as a mediator and moderator in future studies. A longitudinal design could also overcome this limitation, as well as to include a developmental perspective in order to better understand the reciprocal effects between psychological well-being and school performance, and the role of gender and age in this association. Such longitudinal study would also benefit from extending data collection to secondary (15–18-years-old) school level. Furthermore, the inclusion of self-report measures (e.g. Youth Self-Report; Achenbach et al., 2014) could contribute to a more comprehensive approach to the assessment of EBP, particularly IP in adolescents.

### Practical implications

This study stresses the importance to link prevention and mental health promotion with success in school, by highlighting the generalized interplay between school performance and psychological well-being. Busch et al. (2014) reported that most prevention, mental health promotion, or social-emotional learning programs neglect the obvious link with AA. Accordingly, several practical implications of this study for teachers and psychology services in schools must be considered, as success in learning is central in children and adolescents’ psychological functioning. First, the use of broad-band assessment instruments, with multiple informants (e.g. parents, teachers), providing a general perspective on students’ emotional and behavioural problems – such as the ASEBA battery used in this study (Achenbach et al., 2014) – is recommended. Secondly, a preventive approach to EBP in schools should consider a special attention to avoiding repeated academic failure. It is important that teachers try to adjust teaching/learning strategies to individual characteristics of students, their specific difficulties, as recommended by RTI models (Fuchs et al., 2012; Fuchs & Vaughan, 2012). Moreover, a collaborative and integrated approach to academic, social, and emotional development is determinant, as recommended by CASEL model (CASEL, 2020). Finally, a clinical approach with children and adolescents presenting EBP should always consider the relevance of academic performance variables in assessment and intervention.
